# Army Veterinary Service

**Published:** 1901-04

**Authors:** 


### EDITORIALS.

### ARMY VETERINARY SERVICE.

The Fifty-sixth Congress came to an end on March 4th, and the efforts of the eight thousand veterinarians in the United States had still been unable to obtain any betterment of the veterinary service in the United States army. Although, with the approval of the President, the Secretary of War had at the end of the Fifty-fifth Congress indorsed a provision of the Army bill to establish a Veterinary Corps in the army, and large numbers of the most prominent and experienced officers of the army had in the next year declared that an organized veterinary service was needed in the army; and the Senate had in May, 1900, provided for such a service by a majority vote; and the House of Representatives had in December, 1900, concurred in the action of the Senate, and had by a majority vote retained in the Army Reorganization bill, S. 4300, the provision for this service; and the enacting clause of the Army bill, with the veterinary section, constituted a legal act, awaiting only the signature of the President to become a law of the land;

Yet, by some occult influence, a reconsideration was made by the Senate without parliamentary precedent; declared friends of the veterinary cause considered it best to delay the establishment of the veterinary service, and the veterinary section of the Army bill was eliminated from it.

The bill for an improved veterinary service in the army did not pass the last Congress, but the necessity for such a service has attracted wide-spread attention and much interest, and we are confident that it is only a question of a little time until this service will be established. It took seventeen years' constant work before the prejudices in England were overruled and the English Parliament established in the armies of Great Britain a veterinary service on the same basis as those of France, Germany, and the other leading military countries of the world. Even more ignorant prejudices seem to have to be overcome in the United States.

The provisions of the Army Appropriation bill, which passed Congress after the Army Reorganization bill, called for such an array of expenditure for matters which should have expert veterinary services, that we still hoped, until this bill had passed, that further reconsideration might be obtained.

The Army Appropriation bill, H. R. 14,017, provided, as items in its whole amount:

| | |
|---|---|
| 42 veterinarians (civilians) for the cavalry and artillery . | $63,000 |
| 100 veterinarians (civilians) for the Quartermaster's Department . . . . . . . . . . . | 120,000 |
| Such items as: | |
| "Forage in kind for the horses, mules, and oxen of the Quartermaster's Department," "horses of the several regiments of cavalry, the batteries of artillery, mounted infantry, scouts, officers' horses," etc., a portion of . . . | 9,000,000 |
| Such items as: | |
| "Hire of veterinary surgeons, purchase of medicine for horses and mules," "blacksmith's tools and materials, horseshoes and blacksmith's tools for the cavalry service,[1] and for shoeing horses and mules," etc., a portion of . . | 2,400,000 |
| "Purchase of horses for cavalry and artillery" . . . | 750,000 |
| "Purchase and hire of draught and pack animals," etc., a portion of . . . . . . . . . . | 34,000,000 |

One hundred and forty-two civilian veterinarians were provided for at a cost of $183,000.

Six thousand or more horses are to be purchased for the five new regiments of cavalry and for the light artillery.

Horses, mules, and the incidentals for their welfare, in the shape of forage, shoes, medicine, etc., are to be purchased at a cost of $3,000,000 or more.

These purchases are made by the Quartermaster-General.

One hundred of the civilian veterinarians are directly under the Quartermaster-General.

The forty-two veterinarians (also civilians) in the cavalry and artillery are supplied by the Quartermasters.

Of these forty-two veterinarians: twenty (or less, as vacancies exist) are in the old ten regiments of cavalry; ten are to be appointed for the five new cavalry regiments; twelve for the artillery; twenty-two (or more, to fill vacancies) are to be new men.

Under the present system only an inferior class of veterinarians can be secured; and a number of officers in the army wrote the War Department that some of the present veterinarians are not fit, by education and for other reasons, to be commissioned.

With "rank" and proper recognition a better class, who by

[1] Horseshoes for the artillery horses are furnished by the Ordnance Department.

education and other conditions are fitted to be officers, would compete in the examinations.

For furnishing supplies for these 142 veterinarians the Quartermaster-General has no special, expert, or trained officer to supervise the supplies, inspect the professional work, or report upon the results.

This statement seems to present to any common-sense view or practical executive mind a simple, plain business proposition.

Imagine 142 medical officers assigned to various bodies of troops without any system of supply, any method for reporting the result of their work, or any inspection or control beyond that of their immediate commanders, who—experienced commanders as they may be—are not experts in medicine, diagnosis, therapeutics, or the pathology of contagious diseases!

Imagine Senator ——, with large mining interests, or Senator ——, with great agricultural or lumber undertakings, putting 142 miners, farm laborers, or woodsmen to work as individuals, without a foreman or superintendent who would regulate these men's work and report upon the results!

We are looking with interest for the next report of the Committee on Army Legislation of the American Veterinary Medical Association to know in what specific direction our next efforts must be turned, but we are confident of future success.

